# Physalis Calyx seu Fructus relieves chicken intestinal damage to heat via improving the antioxidant ability

**DOI:** 10.3389/fimmu.2024.1536045

**Published:** 2025-01-14

**Authors:** Bin Yin, Changning Juan, Rongling Zhang, Shifa Yang, Haiting Wang, Yueyue Liu, Shikai Song, Zunxiang Yan, Yunpeng Yi, Zengcheng Zhao, Zhongli Huang, Shuqian Lin

**Affiliations:** Poultry Institute, Shandong Academy of Agricultural Science, Jinan, Shandong, China

**Keywords:** Physalis Calyx seu Fructus, heat stress, antioxidant, intestinal damage, chicken

## Abstract

Heat-stress-induced oxidative and inflammatory responses were important factors contributing to chicken intestinal damage. The purpose of this study was based on the antioxidant and anti-inflammatory activities of Physalis Calyx seu Fructus (Jin Deng Long, JDL) to investigate its efficacy and mechanism in relieving chicken heat stress damage. Primary chicken embryo duodenum cells and 90 30-day-old specific-pathogen-free chicken were randomly divided into control and JDL groups to establish heat stress models *in vitro* and *in vivo*. The mitigating effect was assessed through the oxidation-related enzymes and key genes, histopathology, and inflammatory factors. The results demonstrated that 100 µg/mL JDL extract could effectively alleviate heat stress damage to chicken embryo duodenum cells at 42°C. A strong antioxidant capacity of 100 µg/mL JDL extract was shown in the downregulation of LDH (at 5 h, *P* < 0.01) and MDA (at 5 h, *P* < 0.05), in the upregulation of SOD (at 5 and 10 h, *P* < 0.01), CAT (at 5 h, *P* < 0.01), and GSH-PX and T-AOC (at 0 h, *P* < 0.01) as well as in the high transcription level of *NQO1* (at 5 and 10 h, *P* < 0.05) and *HO-1* (at 5 and 10 h, *P* < 0.01). Supplements with 1 and 3 g/kg b.wt, respectively, in the drinking water both suppressed the rise of body temperature and had light pathological lesions of chicken duodenal tissues caused by heat stress at 40 ± 1°C. Accordingly, the chicken of JDL extract groups showed a lower inflammatory response as manifested by a lower level of IL-10 and higher levels of IL-6 and TNF-α and a strong antioxidant capacity characterized by lower level of MDA and higher levels of SOD and GSH-PX in the serum as well as also showed a higher transcription level of *Nrf2*, *NQO1*, and *HO-1* in the duodenal tissues. In conclusion, JDL extract relieved chicken intestinal damage to heat via improving the antioxidant ability and reducing the inflammatory response.

## Introduction

Heat stress refers to the collective physiological responses exhibited by an organism when exposed to high temperatures, and the body function of any animal might be affected by heat stress. Poultry, characterized with thick feathers, skin without sweat glands, vigorous metabolism, and high body temperature, is particularly vulnerable to the effects of high-temperature environments (1). In recent years, despite the fact that standardized and modernized breeding models had been promoted, heat stress continues to be a core issue in the poultry breeding industry during the high-temperature period of summer. Heat stress could lead to a decrease in feed intake, production performance, product quality, and body immunity and could induce disease hazards, ultimately leading to increased mortality (2). When poultry were exposed to a high-temperature environment, the animals might experience loss of appetite and indigestion. The intestine is a crucial site for the absorption and digestion of nutrients, serving as an innate barrier that maintained the balance of the internal environment and blocked the entry of pathogenic bacteria and toxins (3). Impaired intestinal epithelial function not only affected the absorption of nutrients to reduce the production performance but also caused the translocation of pathogenic bacteria and endotoxin within the intestine, thereby triggering infections (4, 5). This was a significant factor causing mortality in poultry.

Research had found that the factors inducing intestinal damage due to heat stress were mainly manifested in three aspects. Firstly, under heat stress conditions, a large amount of blood flowed toward the surface of the body to increase heat dissipation, resulting in a decrease in blood flow to the intestine, which leads to ischemia and hypoxia of intestinal epithelial cells (1). The ischemic and hypoxic conditions of intestinal epithelial cells induced the production of excessive reactive oxygen species (ROS). ROS could initiate lipid peroxidation within the cells, which disrupts the redox homeostasis, causing damage to intestinal tissue and decreasing the regenerative ability of the intestine, which thus fails to timely replace the damaged tissue (6). Secondly, heat stress could activate the hypothalamic–pituitary axis, leading to the secretion of cortisol, proinflammatory cytokines, and ROS. These substances might cause a disruption of intestinal homeostasis through connections between brain–gut axis neurons. This disruption caused abnormal intestinal function, increased inflammatory response, and oxidative stress, which could have adverse effects on the overall health of the organism (5). Thirdly, heat stress could cause a disruption of the intestinal microbiota in poultry, leading to a decrease in the number of beneficial bacteria, such as *Lactobacillus* and *Bifidobacterium*, and an increase in the colonization of pathogens like *Salmonella* and *Clostridium* in the small intestine (5, 7). This adversely affects the growth and production performance and triggers various diseases. Therefore, heat-stress-induced oxidative damage, inflammatory responses to intestinal cells, and alterations in the gut microbiota were the primary factors that led to the impairment of intestinal epithelial function. The development of effective drugs that targeted antioxidant and anti-inflammatory activities and the maintenance of gut microbiota balance had been identified as an effective strategy to alleviate heat-induced intestinal damage.

JDL was a type of dried calyx or calyx with fruits from the plant *Physalis alkekengi* L. var. *franchetii* (Mast.) Makino., which belonged to the family of Solanaceae. It had a cold nature and bitter taste and was effective in clearing heat and detoxifying, benefiting the throat, resolving phlegm, and promoting urination. In ancient medical recipes, it was recorded that a combination of five ounces of JDL fruits, three ounces of amaranth seeds, two ounces of *Iris lactea* rhizome (fried), two ounces of salty elm bark (fried), one ounce of *Bupleurum* root, one ounce of *Scutellaria* root, one ounce of *Trichosanthes* root, and one ounce of *Mesona chinensis* was mixed and ground into powder. When the powder was mixed with honey and formed into pills, taken with a *Magnolia officinalis* decoction, it could be used to treat internal heat in the intestines. Modern research in traditional Chinese medicine chemistry had revealed that the chemical components of JDL primarily encompassed physalin alkaloids, luteolin, flavonoids, steroids, alkaloids, volatile oils, abundant inorganic elements, and polysaccharides (8). In addition, modern pharmacology research had also demonstrated its significant effects in antioxidant, anti-inflammatory, and gut barrier protection (9). Hu conducted pharmacological activity tests on the compounds in the ethanol extract of JDL and discovered that five physalin alkaloids, two sesquiterpenes, two alkaloids, and one flavonoid compound exhibited anti-inflammatory activity (10). Additionally, nine physalin alkaloids, one ergostane compound, one sesquiterpene, two alkaloids, and one flavonoid compound were found to possess antioxidant activity (10). Moniruzzaman also discovered that luteolin glycoside, a compound in JDL, could inhibit the production of ROS in BV2 cells induced by LPS and upregulate the expression of HO-1, thereby exerting its antioxidant effect (11). The pharmacological activities of anti-inflammation and antioxidant compounds were of great significance in alleviating intestinal thermal damage, leading us to speculate that JDL might have a good effect in relieving intestinal thermal damage in chickens.

The Nrf2–ARE signaling pathway was an important endogenous antioxidant defense mechanism, playing a crucial role in maintaining redox balance and protecting cells from oxidative stress. During exposure to heat stress, the Nrf2–ARE signaling pathway could be activated to initiate the expression of various downstream antioxidant enzymes, including NQO1, HO-1, SOD, CAT, and GSH, to protect the organism from oxidative damage. Therefore, this pathway represented a potential therapeutic strategy to target heat stress (12). In this paper, we selected the duodenum to construct *in vivo* and *in vitro* heat stress models and focused on the Nrf2–AER signaling pathway to investigate the mitigating effect of JDL on intestinal heat damage in chickens.

## Materials and methods

### Preparation of JDL extract

A dried calyx of JDL was cut into pieces and placed into a pulverizer for grinding for 30 s. A certain mass of JDL powder was weighed and mixed with 90% ethanol that was eight times its volume. After stirring well, the mixture was sonicated for 20 min (at a power of 250 W and a frequency of 40 kHz), and then the supernatant was collected. Added again to the precipitate was 90% ethanol that was eight times its volume, and the mixture was then sonicated for another 20 min. The supernatant was collected and combined into the first. The supernatant was filtered through a qualitative filter paper and then concentrated to 1 g/mL to obtain the JDL extract. The prepared extract was stored at -20°C, and it should be allowed to warm up to room temperature until all precipitates dissolve before use.

### Isolation and culture of primary chicken embryo duodenum cells

The 16-day-old SPF chicken embryos were disinfected with alcohol, and then the embryo was removed. The duodenum tissue was separated and washed twice in D-Hank’s solution, and then a curved-tip forceps was used to separate the intestinal mucosa. After centrifugation at 800 rpm for 15 min, the supernatant was discarded, and collagenase I with a final concentration of 1 mg/mL was added to the mucosal tissue, and the mixture was digested at 37°C for 30 min. Then, an equal volume of DMEM/F12 medium containing 20% serum was added to stop the digestion. After centrifugation at 800 rpm for 15 min, the cells were collected and resuspended in DMEM/F12 medium containing 20% serum. The cells were passed through a 200-mesh cell strainer and plated at a density of 1 × 10^6^ cells/mL. After culturing in a cell culture incubator for 24 h, the medium was changed and the cells were ready for use.

### Screening for the optimal concentration of JDL extract in mitigating heat stress damage to chicken embryo duodenum cells

Cell viability was employed as a metric to screen the optimal concentration using the Cell Counting Kit-8 (CCK-8, Beyotime, China). Isolated chicken embryo duodenum cells were seeded into 96-well plates, and when the cells reached a confluence of 70%–80%, the media was replaced with fresh media containing JDL extract at 0, 100, 200, 300, 400, 500, 600, and 700 μg/mL. After the cells were cultured at 37°C for 16 h, the medium was replaced by the medium containing 10% CCK-8, and the cells were incubated at 37°C for 2 h to measure the absorbance at 450 nm to determine the safe concentration. Moreover, after incubating the cells with varying concentrations of JDL extract for 16 h, the cells were transferred into an incubator at 42°C with 5% CO_2_ for 5 or 10 h, then the medium was replaced with the medium containing 10% CCK-8, and the cells were incubated at 37°C for 2 h to measure the absorbance at 450 nm to assess the protective effects of the JDL extract.

### *In vitro* chicken embryo duodenal cell heat stress model

**Figure 1:**
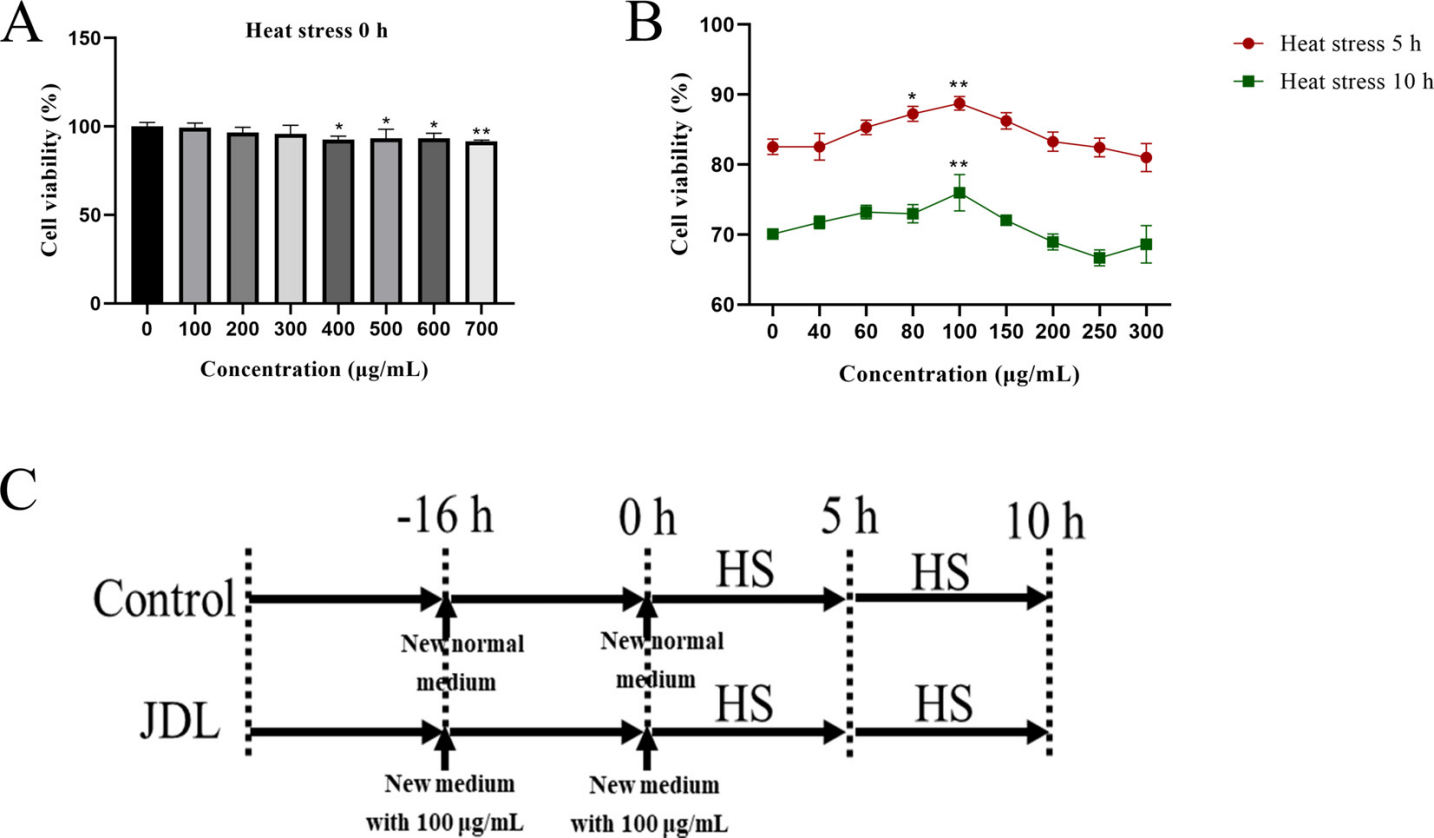
Viability of chicken embryo duodenum cells by CCK 8 kits and experimental design. **(A)** Cell viability with supplement of various concentrations of JDL extract without heat stress. **(B)** Cell viability with supplement of various concentrations of JDL extract following heat stress for 5 and 10 h. **(C)** Experimental design. ^∗^ *P <*0.05 and ^∗∗^ *P <*0.01 were compared with the concentration of 0 µg/mL.

Isolated chicken embryo duodenal cells were randomly divided into a control group and a JDL extract group. When the cell confluence reached 70%–80%, the cell culture medium in the control group was replaced with normal medium, while in the JDL extract group it was replaced with a medium containing 100 μg/mL of JDL extract. After culturing at 37°C for 16 h, the medium was replaced according to the group designation, and the cells were transferred into a cell incubator at 42°C with 5% CO_2_ for 0, 5, and 10 h, respectively (**Figure 1C**). Thereafter, the supernatant and cells were collected separately for the analysis of various indicators.

### Oxidative-damage-related index analysis in chicken embryo duodenum cells

Isolated chicken embryo duodenal cells were seeded in 30-mm dishes and were treated as the *in vitro* heat stress model. After heat stress, the supernatant was harvested for LDH detection (LDH, A020-2-2), and the cells were collected for the quantification of malondialdehyde (MDA, A003-1-2), superoxide dismutase (SOD, A001-3-2), catalase (CAT, A007-1-1), glutathione peroxidase (GSH-PX, A005-1-2), and total antioxidant capacity (T-AOC, A012-5). All of the indicators were processed using kits purchased from Nanjing Jiancheng Bioengineering Institute and according to the instructions.

Dynamic transcriptional levels of *Nrf2*, *HO-1*, and *NQO1* in chicken embryo duodenum cells were analyzed using qPCR, and the primers are shown in **Table 1**. Chicken embryo duodenum cells after heat stress were washed with PBS three times and lysed with RNAiso Plus reagent (TaKaRa, Japan) for the extraction of total RNA, and the RNA was quantified using NanoDrop 2000 (Thermo, USA) by the absorbance at 260 nm and A260/A280 ratio. Then, reverse transcription was performed using Evo M-MLV Reverse Transcription kit to synthesize cDNA, which was quantified with 2xPerfectStartTMGreen qPCR SuperMix kit (AQ601-02-V2, Trans, Beijing). To determine the relative transcription levels of *Nrf2*, *HO-1*, and *NQO1*, the data was normalized against glyceraldehyde-3-phosphate dehydrogenase (GAPDH) and quantified by the comparative Ct (2^−ΔΔCt^) method.

**Table 1:** Real-time PCR primer sequences.

Gene	Forward primer	Reverse primer
Nrf2	5′-AACGCTGAACCACCA-3′	5′-TTCCCAAACTTGCTCTAT-3′
HO-1	5′-CCGCTATTTGGGAGACC-3′	5′-TCAAGGGCATTCATTCG-3′
NQO1	5′-AACCTCTTTCAACCACGCCA-3′	5′-AAGCACTCGGGGTTCTTGAG-3′
GAPDH	5′-TGAAAGTCGGAGTCAACGGAT-3′	5′-ACGCTCCTGGAAGATAGTGAT-3′

### Construction of an *in vivo* heat stress model in chickens and sample collection

**Figure 2:**
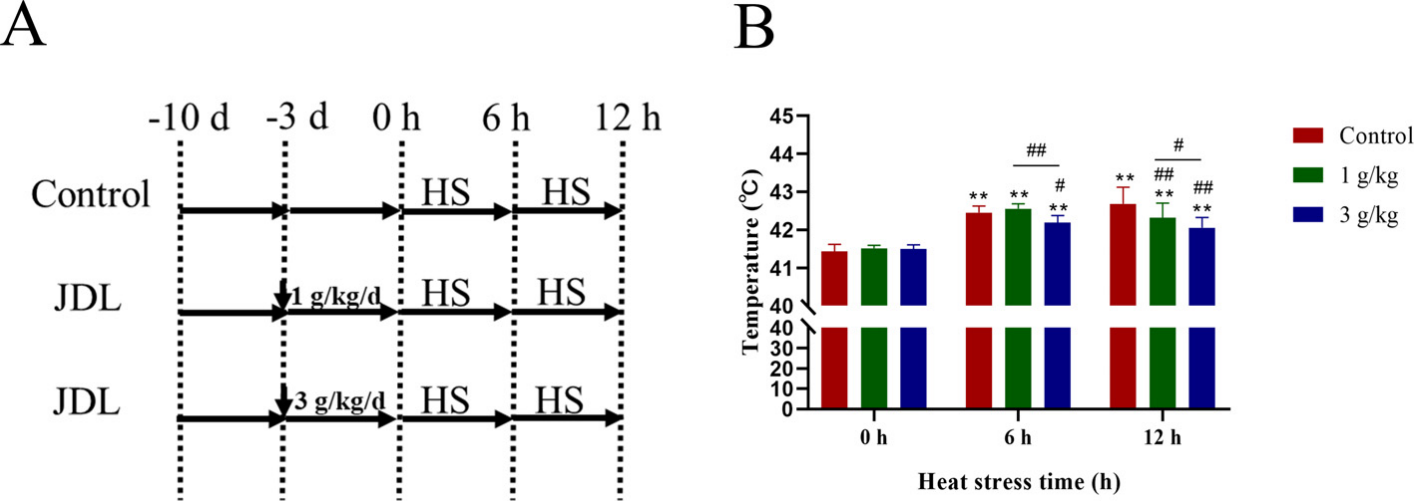
Design of animal experiments and body temperature of SPF chicken. **(A)** Design of animal experiments. **(B)** Body temperature. ^∗^ *P <*0.05 and ^∗∗^ *P <*0.01 were compared with 0 h in the control group; ^#^ *P <*0.05 and ^##^ *P <*0.01 were compared among the control group, 1-g/kg-b.wt-JDL-extract group, and 3-g/kg-b.wt-JDL-extract group under the same conditions.

A total of 90 30-day-old SPF chickens, purchased from Jinan SPFR Industry Science and Technology Ltd., China, were randomly divided into three groups: control group, 1-g/kg-b.wt-JDL group, and 3-g/kg-b.wt-JDL group. During the 3-day drug administration period, chicken in the control group were given normal water, while chicken in the JDL groups were respectively given JDL through their drinking water at a dose of 1 or 3 g/kg b.wt. Then, heat stress (40 ± 1°C and 50% humidity) was triggered for 0, 6, and 12 h with the same drinking with drug administration period (**Figure 2A**). After triggering heat stress, the body temperature was measured, and blood and duodenal tissues were collected immediately. The collected blood samples were immediately centrifuged to separate the serum, and the duodenal tissues were placed in 4% paraformaldehyde or in liquid nitrogen for histopathological or molecular biological analysis. All experimental procedures were conducted strictly adhering to the ethical guidelines and were approved by the Institutional Animal Care and Use Committee of Shandong Academy of Agricultural Sciences, with reference number SAAS-2023-G32.

### Detection of histopathological damage in chicken duodenal tissues

Chicken duodenal tissues were fixed with 4% paraformaldehyde and were sent to Severn Biotech for paraffin sectioning. Pathological changes in the duodenal villi were observed using an optical microscope (OLYMPUS CX33, Japan).

### Analysis of inflammation-related indicators in serum

The levels of cortisol (ml036947), IL-10 (ml059830), IL-6 (ml059839), and TNF-α (ml002790) in chicken serum were measured using kits from Shanghai Enzyme-Linked Biotechnology Co., Ltd., and the measurements were performed following the manufacturer’s recommended instructions.

### Analysis of oxidative-stress-related indicators in serum

The levels of malondialdehyde (MDA, A003-1-2), superoxide dismutase (SOD, A001-3-2), catalase (CAT, A007-1-1), and glutathione peroxidase (GSH-PX, A005-1-2) in chicken serum were measured using kits purchased from Nanjing Jiancheng Bioengineering Institute, and the measurements were performed following the manufacturer’s recommended instructions.

### Dynamic transcriptional levels of *Nrf2*, *HO-1*, and *NQO1* in chicken duodenal tissues

A total of 20 mg of chicken duodenal tissue was weighed, and samples from three to four chickens at the same stress time point are mixed together to form a single sample for detection. After adding 1 mL of RNAplus, this was homogenized at 5,000 rpm under 4°C conditions, and then the extraction of total RNA for qPCR analysis proceeded as the method in chicken embryo duodenum cells.

### Statistical analysis

Experimental data were presented as mean ± standard deviation (SD), and to assess differences among the groups, one-way analysis of variance (ANOVA) and least significant difference (LSD) multiple-comparison test methods were utilized through the SPSS software (version 20). Statistically significant differences were designated as *P* < 0.05 (denoted by ∗ or #), while extremely significant differences were designated as *P* < 0.01 (denoted by ∗∗ or ##).

## Results

### Optimal concentration of JDL extract in primary chicken embryo duodenum cells

The CCK-8 analysis has shown that the concentration of JDL extract within 300 µg/mL had no effect on cell activity (*P* > 0.05), making it a safe concentration for the chicken embryo duodenal cells (**Figure 1A**). When the cells were subjected to heat stress, it was discovered that 100 µg/mL JDL extract could significantly enhance cell activity during heat stress for 5 and 10 h (*P* < 0.01) (**Figure 1B**). This indicated that 100 µg/mL JDL extract could be used as the optimal concentration to alleviate cellular heat stress damage.

### JDL extract relieved oxidative damage in primary chicken embryo duodenum cells induced by heat stress

**Figure 3:**
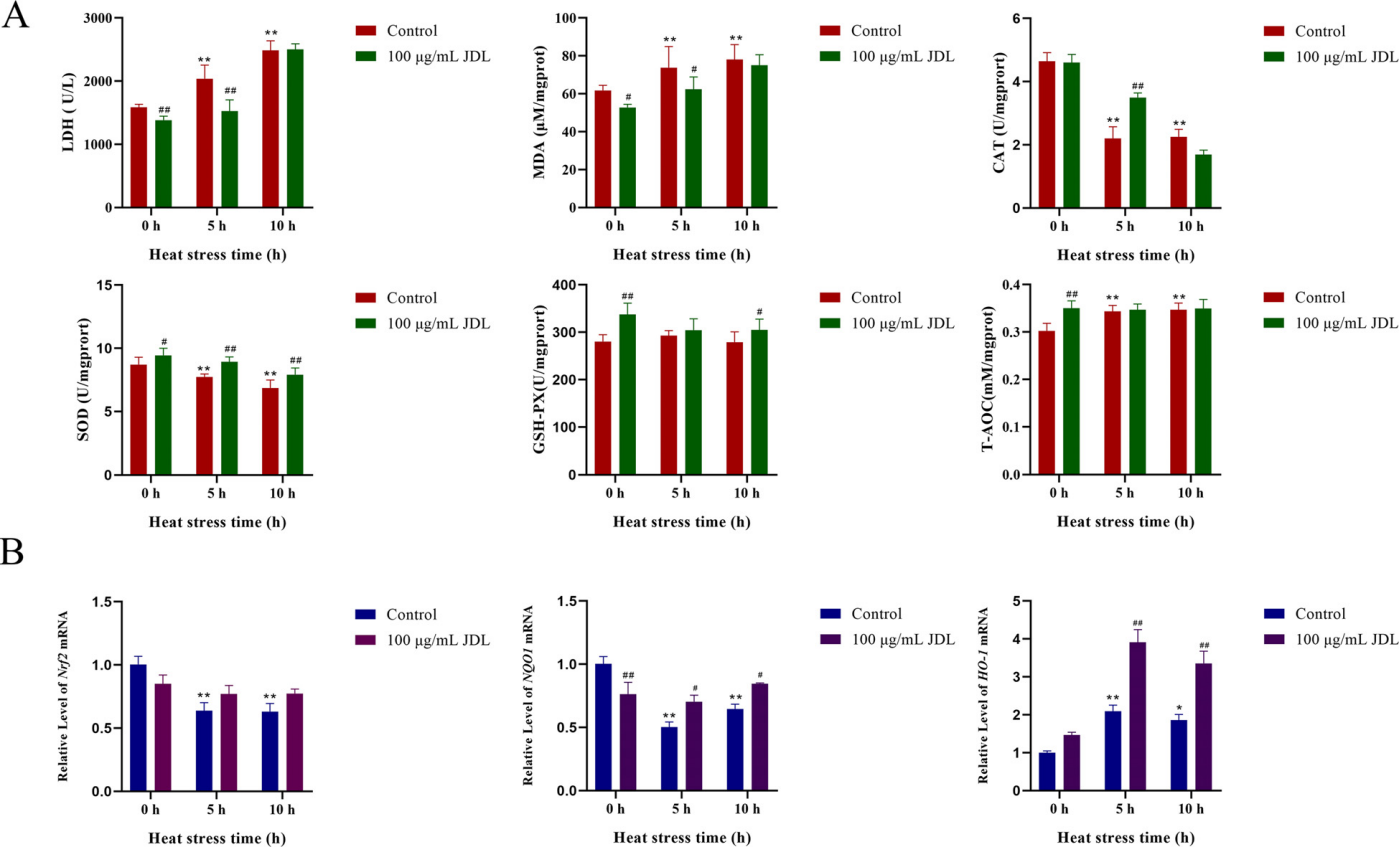
Indicators of oxidative damage to chicken embryo duodenum cells. **(A)** LDH concentration in cell supernatant and MDA, SOD, CAT, GSH-PX, and T-AOC concentration in cells. **(B)** Dynamic transcriptional levels of *Nrf2*, *NQO1*, and *HO-1* in chicken embryo duodenum cells by qPCR. ^∗^ *P <*0.05 and ^∗∗^ *P <*0.01 were compared with 0 h in the control group; ^#^ *P <*0.05 and ^##^ *P <*0.01 were compared between the control and 100-µg/mL-JDL groups under the same conditions.

Oxidative-damage-related indicators in chicken embryo duodenal cells are shown in **Figure 3A**. Among them, LDH activity and MDA content both increased under heat stress condition, which could be inhibited by the supplement with 100 µg/mL of JDL extract. At 0 and 5 h of heat stress, LDH activity decreased by 13% and 25%, respectively, showing extremely significant differences (*P* < 0.01); the content of MDA also showed a significant decrease (*P* < 0.05). Heat stress for both 5 and 10 h could cause a significant downregulation of antioxidant enzymes SOD and CAT (*P* < 0.01), while supplementing with 100 µg/mL JDL extract could significantly increase the activity of SOD during heat stress for 5 and 10 h (*P* < 0.01) as well as the activity of CAT for 5 h (*P* < 0.01). GSH-PX did not change significantly under the influence of heat stress for 5 and 10 h. After the cells were treated with 100 µg/mL JDL extract, the activity of GSH-PX significantly increased by approximately 20% under normal condition (*P* < 0.01) and also increased at 5 and 10 h of heat stress. The total antioxidant capacity (T-AOC) of cells showed a significant upregulation when exposed to heat stress for 5 and 10 h. However, addition of 100 µg/mL JDL extract significantly enhanced the T-AOC level under normal conditions (*P* < 0.01), but there was no difference when the cells were exposed to heat stress.

The dynamic transcription levels of genes related to oxidative damage in chicken embryo duodenum cells are shown in **Figure 3B**. Heat stress significantly caused a decrease in the transcription levels of *Nrf2* and *NQO1* at both 5 and 10 h (*P* < 0.01), while the addition of JDL extract inhibited the downregulation of *Nrf2* (*P* > 0.05) and *NQO1* (*P* < 0.05) induced by heat stress. In the absence of heat stress condition, the addition of JDL extract also reduced the transcription levels of *NQO1* (*P* < 0.01). Heat stress upregulated the transcript level of *HO-1*, which was approximately 1-fold at 5 h (*P* < 0.01), whereas at 10 h there was a slight decrease compared with 5 h, but it was still significantly different from that of 0 h in the control group (*P* < 0.05). The addition of JDL extract could induce the level of *HO-1*, which was upregulated by two times at 5 h of heat stress and upregulated approximately 80% at 10 h, both showing significant differences (*P* < 0.01).

### JDL extract improved the clinical manifestations and body temperature of chicken caused by heat stress

The chickens presented normal behavior within 3 days after the addition of JDL extract before heat stress. When the chicken suffered heat stress for 0.5 h, chicken in all groups exhibited symptoms of heat stress, including open-mouth breathing, increased water intake, decreased food intake, and thin feces. While the heat stress continued for 6 h, chicken in the control group were more sensitive to heat and had more obvious symptoms of lethargy and watery feces compared to the JDL groups. By 12 h of heat stress, chickens in all groups exhibited lethargy and jet-like watery feces, but the chicken in the JDL groups were slightly better. The measurement of body temperature in **Figure 2B** revealed that heat stress caused a significant rise in body temperature by an average of 1°C to 1.2°C at 6 and 12 h (*P* < 0.01). Under normal conditions, supplement with JDL extract at dosages of 1 and 3 g/kg b.wt in the drinking water had no effect on the body temperature of chicken, with an average body temperature of 41.4°C. When the chicken were exposed to heat stress, compared to the control group at the same time points, the body temperature of the 1-g/kg-b.wt-JDL group decreased by 0.37°C at 12 h (*P* < 0.01), and the body temperature of the 3-g/kg-b.wt-JDL group was found to be 0.26°C lower at 6 h (*P* < 0.05) and 0.63°C lower at 12 h (*P* < 0.01).

### JDL extract alleviated the pathological damage in chicken duodenal tissues

**Figure 4:**
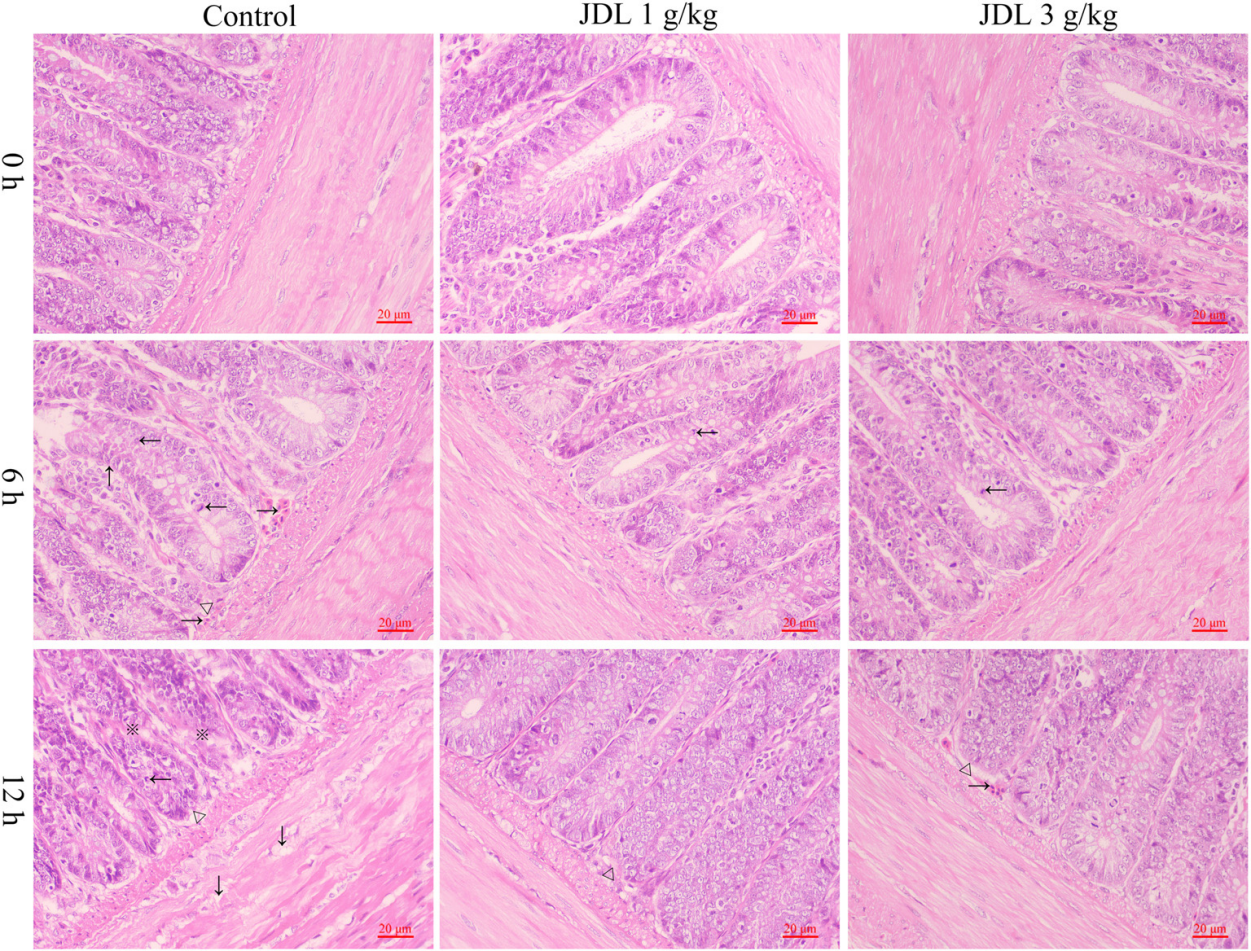
Histopathological damage to chicken duodenal tissues by HE staining. Chicken duodenal tissues, HE staining, 1 bar = 20 μm. Capillary hyperemia (→), crypt distortion (↑), crypt moving upward (△), lymphoid aggregates (←), edema (↓), crypt branching (※).

Heat stress for 6 h could lead to capillary hyperemia in the submucosa of the chicken duodenum, crypt distortion, crypt moving upward, and lymphoid aggregates within the crypt. When heat stress continued for 12 h, obvious edema occurred in the muscularis of the chicken duodenum, along with crypt branching, crypt moving upward, and lymphoid aggregates within the crypt. Addition of 1 or 3 g/kg b.wt of JDL extract both improved heat-stress-induced duodenal pathology, with only lymphoid aggregates within the crypt at 6 h of heat stress and only capillary hyperemia in the submucosa of the chicken duodenum and crypt moving upward at 12 h of heat stress (**Figure 4**).

### JDL improved the inflammation-related indicators in serum

**Figure 5:**
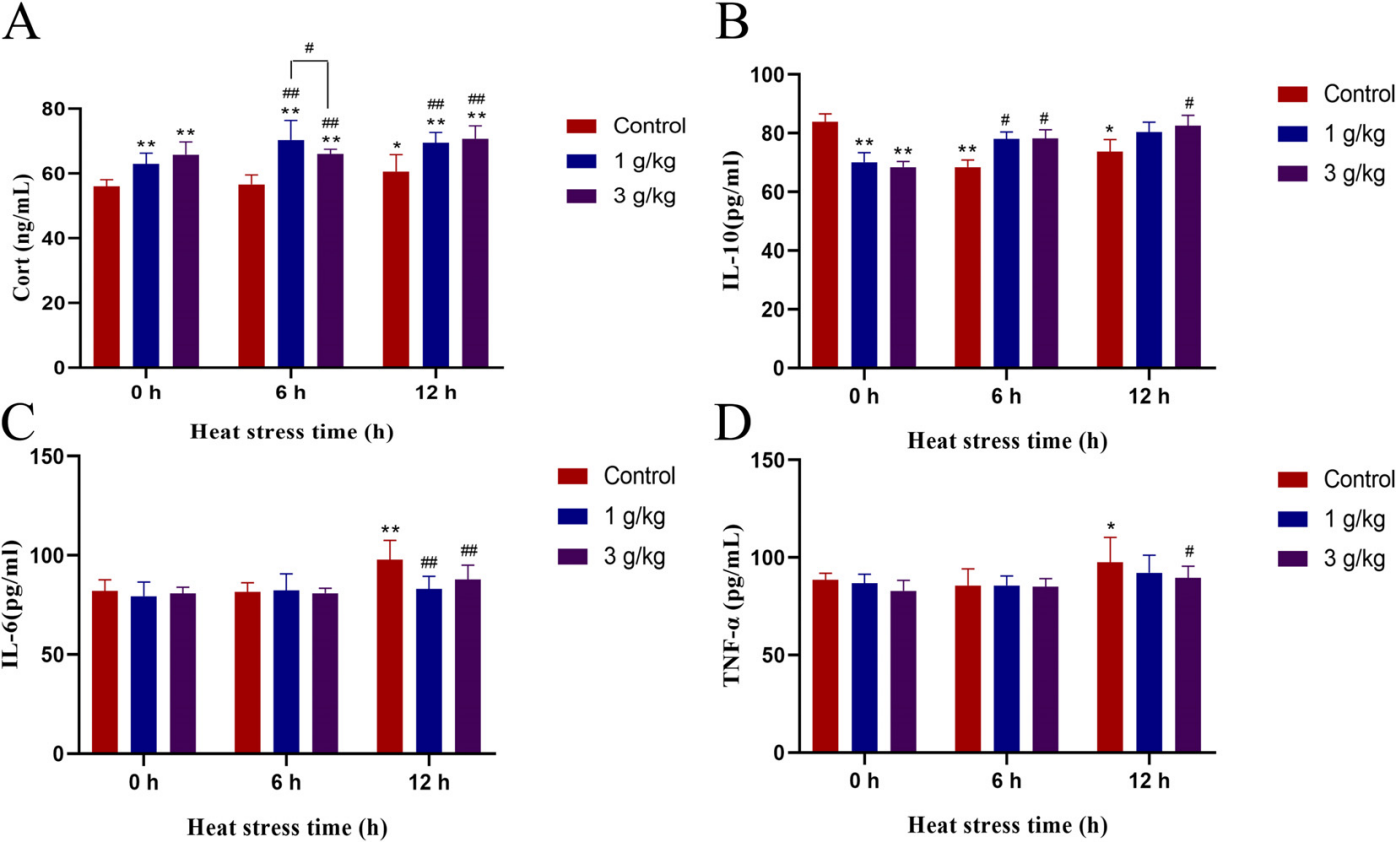
Indicators of inflammation in chicken serum by ELISA. **(A)** Cortisol concentration in chicken serum. **(B)** IL-10 concentration in chicken serum. **(C)** IL-6 concentration in chicken serum. **(D)** TNF-α concentration in chicken serum. ^∗^ *P <*0.05 and ^∗∗^ *P <*0.01 were compared with 0 h in the control group; ^#^ *P <*0.05 and ^##^ *P <*0.01 were compared among the control group, 1-g/kg-b.wt-JDL-extract group, and 3-g/kg-b.wt-JDL-extract group under the same conditions.

Cortisol and IL-10 were important indicators to evaluate the anti-inflammatory level. Heat stress induced a decrease in serum cortisol levels in chickens at 6 h, but there was no significant difference, whereas a significant increase occurred at 12 h (*P* < 0.05) (**Figure 5A**). The addition of 1 or 3 g/kg b.wt of JDL extract both resulted in a significant increase of serum cortisol levels at 0, 6, and 12 h of heat stress (*P* < 0.01), with an increase extent of approximately 12% to 22%. The level of IL-10 in the serum showed a significant decrease during heat stress at 6 h (*P* < 0.01) and 12 h (*P* < 0.05) (**Figure 5B**). Addition of 1 g/kg b.wt of JDL extract to drinking water significantly upregulated the level of IL-10 at 6 h of heat stress (*P* < 0.05), while adding 3 g/kg b.wt of JDL extract to drinking water upregulated the IL-10 levels at 6 and 12 h of heat stress (*P* < 0.05). TNF-α and IL-6 were important indicators to evaluate inflammatory injury. Heat stress could lead to the increase of IL-6 and TNF-α within the serum, respectively showing significant (*P* < 0.01) and extremely significant (*P* < 0.05) increase at 12 h of heat stress (**Figures 5C, D**). Addition of 1 g/kg b.wt of JDL extract inhibited the increase in IL-6 levels caused by 12 h of heat stress (*P* < 0.01). Meanwhile, addition of 3 g/kg b.wt of JDL extract suppressed the upregulation of TNF-α (*P* < 0.05) and IL-6 (*P* < 0.01) caused by heat stress for 12 h.

### JDL extract relieved oxidative damage in chicken induced by heat stress

**Figure 6:**
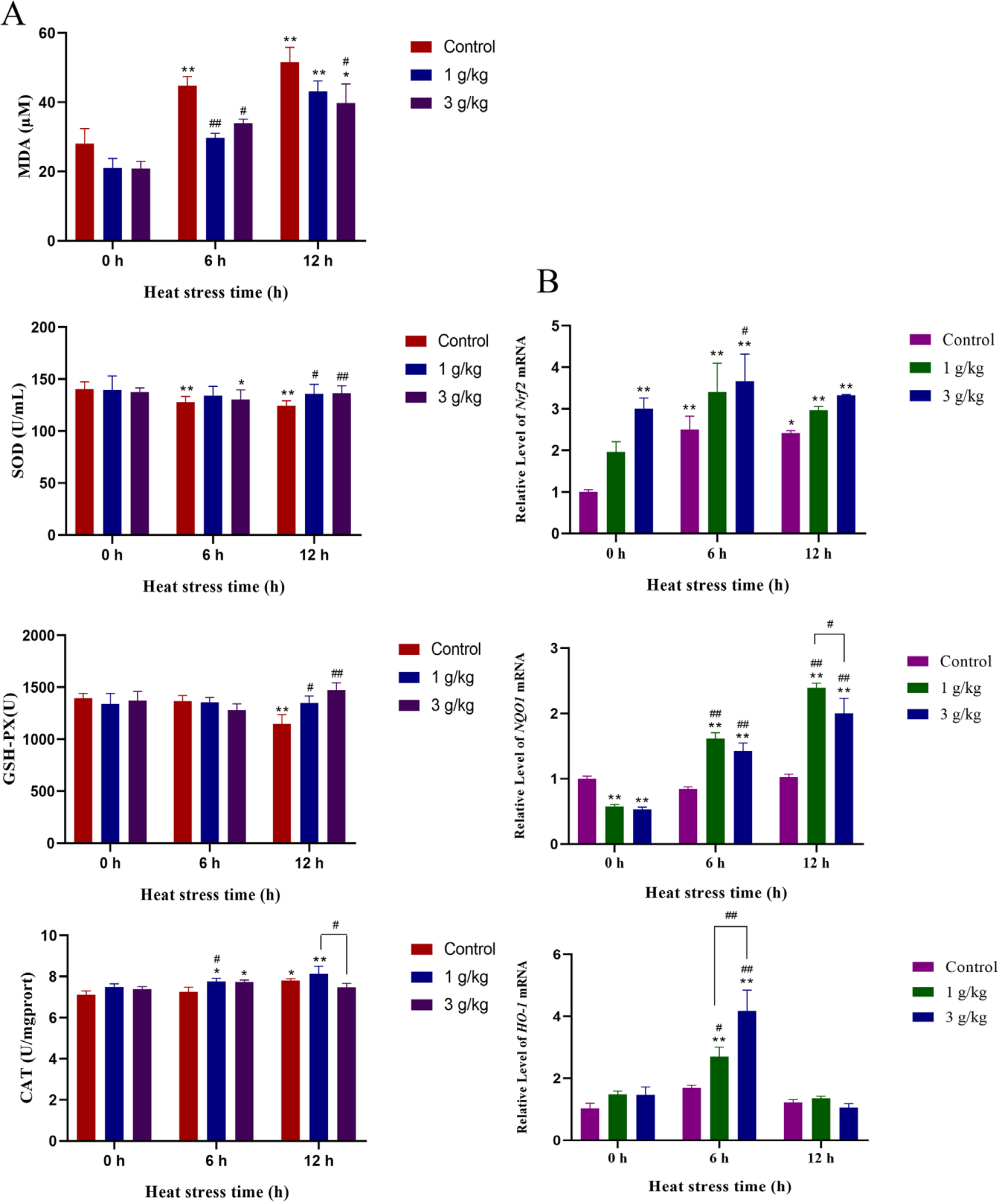
Indicators related to oxidative damage in chicken serum. **(A)** MDA, SOD, GSH-PX, and CAT concentration in chicken serum. **(B)** Dynamic transcriptional levels of *Nrf2*, *NQO1*, and *HO-1* in chicken duodenal tissues by qPCR ^∗^ *P <*0.05 and ^∗∗^ *P <*0.01 were compared with 0 h in the control group; ^#^ *P <*0.05 and ^##^ *P <*0.01 were compared among the control group, 1-g/kg-b.wt-JDL-extract group, and 3-g/kg-b.wt-JDL-extract group under the same conditions.

Antioxidant enzyme activities in chicken serum are shown in **Figure 6A**. Heat stress induced a significant increase in the level of MDA in the serum, with increases of 59% and 84% at 6 and 12 h, respectively, showing a very significant difference (*P* < 0.01). The addition of JDL extract inhibited the increase of MDA level caused by heat stress. Among them, the MDA level in the 1-g/kg-b.wt group decreased by 34% at 6 h, showing a significant difference (*P* < 0.01), while the 3-g/kg-b.wt group decreased by 24% and 23% at 6 and 12 h, respectively, both showing significant differences (*P* < 0.05). Heat stress induced a persistent decrease in SOD and GSH-PX activities in chicken serum, with SOD already showing a significant decrease at 6 h (*P* < 0.01) and GSH-PX at 12 h (*P* < 0.01). The change in CAT activity was opposite to that of SOD and GSH-PX when the chicken suffered heat stress and showed a significant increase at 12 h (*P* < 0.05). Under the same condition of heat stress for 12 h, compared with the control group, the levels of SOD and GSH-PX in the 1-g/kg-b.wt-dose group were significantly increased (*P* < 0.05), while the levels of SOD and GSH-PX in the 3-g/kg-b.wt group were extremely significantly increased (*P* < 0.01).

The dynamic transcription levels of genes related to oxidative damage in chicken duodenal tissues are shown in **Figure 6B**. Heat stress induced a decrease in the level of *Nrf2* at 6 h (*P* < 0.01) and 12 h (*P* < 0.05), while at the same time point of heat stress, the addition of JDL extract further upregulated the level of *Nrf2*. Specifically, the 3-g/kg-b.wt-JDL group exhibited extremely significant differences at 0 h (*P* < 0.01) and significant differences at 6 h (*P* < 0.05) of heat stress. Heat stress had little impact on the levels of *HO-1* and *NQO1* at 6 and 12 h. The addition of 1 and 3 g/kg b.wt of JDL extract could upregulate the level of *NQO1* at 6 and 12 h of heat stress, and both showed highly significant differences, whereas the level of *HO-1* had only shown a difference at 6 h of heat stress, with 1 g/kg b.wt (*P* < 0.05) and 3 g/kg b.wt (*P* < 0.01).

## Discussion

Heat stress could lead to an increase in body core temperature and also result in a decrease in feed utilization, metabolic efficiency, hormone levels, and immune system function (2, 13). The intestinal tract played a crucial role in maintaining normal physiology and immunity in chickens. The duodenum, as the starting point of the intestinal system, was also one of the tissues that have undergone significant thermal damage under heat stress conditions. Understanding the activation of the duodenal antioxidant defense system and identifying methods to enhance its defensive capabilities could help improve the overall health and production performance of chickens exposed to heat stress. Increasing evidence have shown that the root cause of heat-stress-induced damage to the organism lies in the gastrointestinal tract (14). Intestine was the most important organ for digestion, absorption, and immunity. Heat stress could damage the intestinal villus structure, resulting in a rough and uneven surface of the intestinal villi and even rupture of local intestinal villi (15, 16). Research found that heat stress caused damage to the intestinal barrier, and its damage could lead to the entry of pathogenic bacteria or toxins from the intestine into the circulatory system, resulting in local and systemic inflammatory responses, which can activate the immune system and other negative effects (17). Therefore, finding methods to alleviate intestinal heat damage was an effective strategy to reduce heat loss.

Chinese traditional medicine was characterized by its natural properties, low toxicity, no drug residues, low risk of drug resistance, and no pollution to the ecological environment. It worked in a mode of “multiple components–multiple targets–multiple pharmacological effects”. Multiple studies had found that Chinese traditional medicine could improve immunity, antioxidant capacity, and intestinal microbiota as well as inhibit oxidative/inflammatory pathways (15, 18–20). All of these functions provide animals with the ability to resist stress stimuli (19). Studies had shown that *Artemisia* spp., *Olea europaea* L., *Silybum marianum*, *Foeniculum vulgare* Mill., *Thymus vulgaris*, and *Salvia rosmarinus* could alleviate heat stress damage (21). JDL was a type of dried calyx or calyx with fruits from the plant *Physalis alkekengi* L. var. *franchetii* (Mast.) Makino. The analysis of its chemical components and activities revealed multiple compounds exhibiting antioxidant and anti-inflammatory activities (10). Therefore, in this study, JDL was selected to investigate its function in alleviating heat stress. According to the Chinese Veterinary Pharmacopoeia, the dosages of JDL were designed as 1 and 3 g/kg b.wt.

MDA was one of the primary products of lipid peroxidation, and its level could directly reflect the degree of lipid peroxidation. SOD, CAT, and GSH-PX were crucial components of the body’s antioxidant system and served as downstream targets of the Nrf2–ARE signaling pathway. Heat stress resulted in a decrease in SOD levels in the jejunal tissue of broiler chickens, accompanied by an increase in MDA levels in serum and jejunal tissues (15). Literature (22–24) indicated that heat stress induced the production of excessive ROS in the cellular system, leading to significant decreases in multiple antioxidant elements such as glutathione peroxidase (GPx), superoxide dismutase (SOD), and catalase (CAT), as well as decreased the total antioxidant capacity and Nrf2 levels (25, 26). The Nrf2-mediated antioxidant response maintained cellular redox homeostasis by inducing the transcription of multiple cytoprotective genes (27). Resveratrol significantly activated the SIRT1–Nrf1/Nrf2 signaling pathway, increasing the activity of antioxidant enzymes such as SOD and CAT (15). These were consistent with our research that JDL extract upregulated the levels of *Nrf2*, *HO-1*, and *NQO1* as well as the activities of antioxidant enzymes such as SOD, GPx, and CAT, thereby enhancing the antioxidant capacity of duodenal cells and mitigating heat damage.

IL-10 was a crucial anti-inflammatory factor. TNF-α was a pro-inflammatory mediator; its upregulation increased the intestinal permeability (28). IL-6 affected the tight junctions in the gastrointestinal tract (28). *Elephantopus scaber* L. alleviated inflammation caused by heat stress as evidenced by its ability to reduce the TNF-α and IL-6 levels (29). Betaine supplement upregulated the serum content of IL-10 and alleviated heat-stress-induced intestinal damage by inhibiting the inflammatory response (30, 31). These abovementioned results were consistent with our conclusion that JDL extract downregulated the levels of TNF-α and IL-6 and upregulated IL-10 to alleviate heat-stress-induced inflammation.

## Conclusion

In summary, this study established *in vivo* and *in vitro* heat stress models of intestinal epithelial cells and demonstrated that the addition of JDL extract could effectively alleviate heat-stress-induced intestinal injury. This protective effect was associated with the improvement of cellular antioxidant capacity.

## Data Availability

The datasets presented in this study can be found in online repositories. The names of the repository/repositories and accession number(s) can be found in the article/supplementary material.
